# Does the insurance status influence in-hospital outcome? A retrospective assessment in 30,175 surgical trauma patients in Switzerland

**DOI:** 10.1007/s00068-021-01689-x

**Published:** 2021-05-28

**Authors:** Lukas Funke, Claudio Canal, Franziska Ziegenhain, Hans-Christoph Pape, Valentin Neuhaus

**Affiliations:** Division of Trauma Surgery, Department of Traumatology, University Hospital Zurich, University of Zurich, Raemistrasse 100, CH-8091 Zurich, Switzerland

**Keywords:** Insurance status, Orthopaedic trauma, Outcome

## Abstract

**Introduction:**

There has been growing evidence in trauma literature that differences in insurance status lead to inequality in treatment and outcome. Most studies comparing uninsured to insured patients were done in the USA. We sought to gain further insights into differences in the outcomes of trauma patients in a healthcare system with mandatory public health coverage by comparing publicly versus privately insured patients.

**Methods:**

We used a prospective national quality assessment database from the *Arbeitsgemeinschaft für Qualitätssicherung in der Chirurgie* (AQC). More than 80 surgical departments in Switzerland are part of this quality program. We included all patients in the AQC database with any S- or T-code diagnosis according to the International Classification of Diseases ICD-10 (any injuries) who were treated during the 11-year period of 2004–2014. Missing insurance status information was an exclusion criterion. In total, 30,175 patients were included for analysis. The primary outcome was in-hospital mortality. Secondary outcomes included overall and intra- and postoperative complications. Bi- and multivariate analyses were performed, adjusted for insurance status, age, sex, American Society of Anesthesiologists (ASA) physical status category, type of injury, and surgeon’s level of experience.

**Results:**

In total, 76.8% (*n* = 23,196) of the patients were publicly insured. Patients with public insurance were significantly younger (*p* < 0.001), more often male (*p* < 0.001), and in better general health according to the ASA physical status category (*p* < 0.001). Length of pre- and postoperative stay and the number of operations per case were similar in the two groups. Patients with public insurance had a lower mortality rate (1.3% vs. 1.9%, *p* < 0.001), but after adjusting for confounders, insurance status was not a predictor of mortality. Overall complication rates were significantly higher for publicly insured patients (8.4% vs. 6.2%, *p* < 0.001), and after adjusting for confounders, insurance status was identified as an independent risk factor for overall complications (*p* < 0.001).

**Conclusion:**

Differences exist with respect to patient and procedural characteristics: publicly insured patients were younger, more often male, and scored better on ASA physical status. Insurance status seems not to be a predictor for fatal outcome after trauma, although it is associated with complications.

## Introduction

There has been growing evidence in medicine that differences in insurance status lead to inequality regarding the outcome of various diseases. For example, uninsured and Medicaid populations were at greater risk of developing postoperative complications and dying, compared to privately insured patients, after surgery for colorectal carcinoma [1, 2]. Similarly, patients with non-private insurance had higher morbidity and mortality rates after radical prostatectomy compared with privately insured patients [3]. In addition, being uninsured led to a poorer prognosis in oral cavity cancer attributed to an advanced stage of the disease or treatment in less specialised clinics [4]. All these studies were done in the United States and therefore focused on comparing uninsured with insured patients.

Do these differences in treatment and outcome also exist in trauma care? Trauma care often follows protocols [5] and has a rather standardized system of care. Thus, treatment is often initiated directly without the patient’s insurance status being known, so there should be fewer insurance-biased treatment choices compared to other medical fields. Nevertheless, a growing body of trauma literature suggests a relationship between insurance status and the outcome of trauma. Nearly all studies have shown a higher mortality rate for uninsured patients [6–13].

In Switzerland, the Federal Health Insurance Act of 1996 made health insurance compulsory for everyone and introduced a standard benefits package [14]. Services not included in this package can be acquired through additional private insurance. Such services include, among others, free choice of hospital, hospital stays in single rooms, and exclusive treatment from senior physicians.

The question of whether these differences between public and private insurance impact the treatment and outcome of trauma patients has so far been studied very little in countries with compulsory health insurance. An initial study in a Swiss level-one trauma centre found some evidence of an association of insurance status with treatment and outcome of trauma patients [15]. The relationship between insurance status and treatment as well as outcome is likely very complex. To gain further insights, we examined a large cohort treated in 70 different surgical institutions in Switzerland to see whether the level of insurance is associated with variables like mortality, complications, and length of stay.

## Methods

### Study design

The data for this retrospective study were gathered using a prospective surgical registry from the “Working Group for Quality Assurance in Surgery” (“*Arbeitsgemeinschaft für Qualitätssicherung in der Chirurgie*,” AQC). The AQC is an association of chief and attending physicians whose purpose is to collect data on various interventions and surgical diseases and injuries [16]. Through scientific analyses of the collected data, important insights are gained for quality assurance in surgery. Since 1995, the tool has been successfully used to ensure more transparency and a resulting increase in quality in Swiss surgery [17]. More than 80 Swiss surgical departments are part of the AQC (85% of all Swiss surgical departments). They register their surgical cases online using the AdjumedCollect tool [18].

The AQC database is based on a two-part questionnaire. The first part collects information concerning the operation(s), including type of operation (outpatient vs. inpatient and elective vs. emergent), number of operations (per hospitalisation), Swiss surgical procedure classification (CHOP) [19], level of surgeon’s experience (resident, junior or senior consultant), duration of the procedure, need for transfusion(s), anaesthesia as well as intra- or postoperative complications (directly related to the procedure). Patient information is collected in the second part of the questionnaire, which includes age, sex, admission type (emergency, planned), level of insurance, diagnosis codes according to ICD-10 [20], the American Society of Anaesthesiologists (ASA) physical status category [21], thromboembolism prophylaxis or therapy, hospitalisation duration, ICU admission, general/overall complications (e.g., pneumonia), type of discharge (lethal outcome included).

The AQC registry contains only anonymised health data and therefore complies with the authorisation requirements of the local cantonal ethical review board (Zurich, Switzerland) without being subject to approval [22].

### Study subjects

Included were 31,692 patients found in the AQC Register between 2004 and 2014 with an S or T diagnosis code according to the ICD-10 [20]. The ICD-10 is an international classification of diseases and related health problems. It is divided into 22 chapters (e.g., Chapter 9 is about diseases of the circulatory system). Chapter 19 (S- and T-codes) relates to injury, poisoning, and certain other consequences of external causes. S-codes are mainly based on anatomic areas (e.g., head, neck, chest). T-codes represent mainly multiple body injuries, burns, and certain early complications. Missing information about insurance status was an exclusion criterion and was applied to 1,517 patients. Ultimately, 30,175 patients were analysed.

### Variables and outcome measures

The aim was to investigate outcome differences due to insurance status. Therefore, the level of insurance was stratified into public and private; the latter also included semiprivate.

Our primary outcome measurement was in-hospital mortality. Secondary outcome measurements included overall, intra- and postoperative complications, as well as length of stay.

Possible confounders, such as age, sex, ASA physical status, type of injury, experience level of surgeon, surgery and ICU duration, and thromboembolism and antibiotic prophylaxis were assessed. Due to missing data, we could not assess the type of procedure as a possible predictor/confounder for mortality and complications.

### Statistical analysis

Analysis between groups of categorical data was done using the Chi square or Fisher exact test and presented as the number of patients and percentages. The unpaired *t* test was used to compare two groups of continuous data, and results are presented as means ± standard deviation.

Age, sex, ASA physical status, type of injury, experience level of surgeon, surgery and ICU durations, and thromboembolism and antibiotic prophylaxis were chosen as potential risk factors for mortality and complications. These risk factors were evaluated as confounders in a stepwise backward likelihood logistic regression analysis. A *p* value of < 0.001 was considered statistically significant; a *p* value of 0.001–0.049 showed suggestive evidence. The SPSS Statistics program (version 25, IBM software, Armonk, NY) was used to analyse the data.

## Results

### Demographic data

This study included a total of 30,175 injured patients with a mean age of 54 years treated in 70 Swiss hospitals between 1 January 2004 and 31 December 2014; 50.2% were male. The most common injuries included knee and lower leg (29.6%), elbow and forearm (17.0%), and hip and thigh injuries (16.9%).

### Association of insurance status and patient/procedural characteristics

In total, 76.8% (*n* = 23,196) of the patients were publicly insured. Patients with public insurance were significantly younger (53 vs. 61 years), more often male (52.7% vs. 41.8%), and healthier (according to ASA physical status category) than privately insured patients (*p* < 0.001) (Table 1).

**Table 1:** Public vs. Private, Patient Characteristics

Parameter	Public (*n* = 23,196)		Private (*n* = 6979)		*p* value
	*n*	%	*n*	%	
Age (years)					
Mean ± SD	53 ± 24		61 ± 20		< 0.001
Sex					
Male	12,235	52.7	2919	41.8	< 0.001
Female	10,961	47.3	4060	58.2	
ASA					
I (healthy)	9667	43.2	2105	33.5	< 0.001
II (mild systemic disease)	8827	39.5	2970	47.3	
III (severe systemic disease)	3614	16.2	1140	18.2	
IV (severe systemic disease with constant threat to life)	249	1.1	62	1.0	
V (moribund person who is not expected to survive without the operation)	9	0.0	2	0.0	
Admission type					
Emergency	16,006	69.0	4679	69.8	n.s
Registered, planned	7115	30.7	2010	30.0	
Other	72	0.3	15	0.2	
Diagnosis (ICD-10)					
S00-S09: Head Injury	227	1.0	87	1.2	< 0.001
S10-S19: Neck Injury	21	0.1	7	0.1	
S20-S29: Thoracic Injury	239	1.0	89	1.3	
S30-S39: Abdominal, lumbosacral, LWS, pelvis injury	359	1.5	115	1.6	
S40-S49: Shoulder and upper arm injury	2831	12.1	885	12.7	
S50-S59: Elbow and forearm injury	3935	16.9	1188	17.0	
S60-S69: Wrist and hand injury	1429	6.1	154	2.2	
S70-S79: Hip and thigh injury	3835	16.4	1265	18.1	
S80-S89: Knee and lower leg injury	6783	29.1	2160	30.9	
S90-S99: Ankle and foot injury	783	3.4	161	2.3	
T00-T98: Any T code	2883	12.4	870	12.5	

*SD* Standard Deviation, *ASA* American Society of Anesthesiologists Classification system, *n.s*. not significant, *ICD* International Classification of Diseases

Patients with public insurance were operated by less experienced surgeons (*p* < 0.001) and had longer procedural durations (*p* < 0.001). They also stayed longer in the intensive care unit (*p* = 0.049) and were intubated more often (*p* = 0.038). In addition, the public cohort received lower thromboembolism prophylaxis (*p* < 0.001) and prophylactic antibiotics (*p* < 0.001) (Table 2).

**Table 2:** Public vs. Private, Procedure Characteristics

Parameter	Public		Private		*p* value
	*n*	%	*n*	%	
Surgeon class					
Senior consultant, attending surgeon	9431	40.8	4651	71.7	< 0.001
Junior consultant	8874	38.4	1384	21.3	
Resident	4824	20.9	450	6.9	
Duration surgery (minutes)					
Mean ± SD	94 ± 128		82 ± 93		< 0.001
Duration ICU (hours)					
Mean ± SD	3 ± 33		2 ± 23		0.049
Need for intubation					
No	883	3.8	227	3.3	0.038
Thromboembolism prophylaxis					
No	3673	15.9	545	8.1	< 0.001
Yes	18,111	78.2	5717	84.7	
Anticoagulation	1374	5.9	488	7.2	
Antibiotics					
No	4894	21.2	1092	16.8	< 0.001
Prophylactic before incision	15,426	66.8	4771	73.3	
Prophylactic after incision	765	3.3	179	2.7	
Antibiotic therapy	2024	8.8	471	7.2	

*SD* Standard Deviation, *ICU* Intensive Care Unit

### Outcome

Pre- and postoperative length of stay, number of operations per case, and postoperative complications were similar for both groups.

Publicly insured patients had a lower mortality rate (1.3% vs. 1.9%; *p* < 0.001). Public insurance status was not revealed as a risk factor for mortality in multivariate analysis.

Publicly insured patients had a higher chance of intraoperative complications (0.6% vs. 0.4%; *p* = 0.045). The most common intraoperative complications were: (1) other complications, (2) fracture, (3) artery lesion, (4) nerve lesion, and (5) tendon lesion. In addition, overall complications were seen more often in the public cohort (8.4% vs. 6.2%; *p* < 0.001). In multivariate analysis for any complication, public insurance status remained an independent risk factor for complications (OR 1.78, 95% CI 1.49–2.14, *p* < 0.001). The public cohort was discharged to a rehabilitation clinic less often (6.9% vs. 11.4%; *p* < 0.001) (Tables 3,4,5).

**Table 3:** Public vs. Private, Outcome

Parameter	Public		Private		*p* value
	*n*	%	*n*	%	
Intraoperative complications	137	0.6	27	0.4	0.045
Postoperative complications	751	3.2	218	3.1	n.s
Overall complications	984	8.4	234	6.2	< 0.001
Mortality	305	1.3	135	1.9	< 0.001
Discharge					
Deceased	305	1.3	135	1.9	< 0.001
At home	18,554	80.2	5159	77.7	
Nursing home	1008	4.4	219	3.3	
Old people's home	751	3.2	186	2.8	
Psychiatry	92	0.4	11	0.2	
Rehabilitation clinic	1603	6.9	755	11.4	
Other hospital	390	1.7	89	1.3	
Prison	12	0.1	3	0.0	
Others	205	0.9	48	0.7	
Unknown	160	0.7	21	0.3	
Transfer to other ward	57	0.2	15	0.2	
LOS preoperative (days)					
Mean ± SD	2 ± 10		2 ± 26		n.s
LOS postoperative (days)					
Mean ± SD	7 ± 12		8 ± 27		n.s
Number of operations per case					
Mean ± SD	1 ± 1		1 ± 1		n.s

*n.s.:*not significant, *LOS* Lenght of Stay, *SD* Standard Deviation

**Table 4:** Predictors for Mortality

Parameter	Sig	OR	95% CI	
			Lower	Upper
ASA V (vs. ASA I)	< 0.001	20.943	4.406	99.554
ASA IV (vs. ASA I)	< 0.001	12.412	6.036	25.520
Head injury (vs. any T code)	< 0.001	4.605	2.021	10.496
ASA III (vs. ASA I)	0.005	2.582	1.336	4.989
Intubation yes	< 0.001	2.206	1.454	3.345
Sex (male)	< 0.001	2.072	1.562	2.747
Hip and thigh injury (vs. any T code)	0.010	1.770	1.146	2.735
Abdominal, lumbosacral, LWS, pelvis injury (vs. any T code)	n.s	1.461	0.633	3.372
Public insurance (vs. private)	n.s	1.416	0.991	2.023
Junior consultant (vs. senior consultant)	n.s	1.273	0.931	1.740
Antibiotic prophylaxis before incision (vs. no antibiotics)	n.s	1.249	0.734	2.124
Antibiotics as therapy (vs. no antibiotics)	n.s	1.166	0.626	2.175
Number of operations per case	0.028	1.139	1.014	1.279
Thoracic injury (vs. any T code)	n.s	1.077	0.384	3.019
Age	< 0.001	1.060	1.046	1.074
Resident (vs. senior consultant)	n.s	1.009	0.677	1.503
LOS preoperative	n.s	1.006	0.980	1.034
ICU duration	< 0.001	1.004	1.003	1.006
Duration surgery	0.024	0.998	0.997	1.000
Wrist and hand injury (vs. any T code)	n.s	0.983	0.288	3.357
ASA II (vs. ASA I)	n.s	0.750	0.377	1.492
Thromboembolism prophylaxis (vs. no thromboembolism prophylaxis)	n.s	0.645	0.368	1.130
Anticoagulation (vs. no thromboembolism prophylaxis)	0.040	0.489	0.247	0.968
Ankle and foot injury (vs. any T code)	n.s	0.420	0.056	3.131
Elbow and forearm injury (vs. any T code)	0.003	0.235	0.089	0.619
Antibiotic prophylaxis after incision (vs. no antibiotics)	0.042	0.206	0.045	0.945
Shoulder and upper arm injury (vs. any T code)	0.002	0.104	0.024	0.441
Knee and lower leg injury (vs. any T code)	0.001	0.037	0.005	0.272

*ASA* American Society of Anesthesiologists classification system, *n.s.* not significant, *LOS* Length Of Stay, *ICU* Intensive Care Unit

**Table 5:** Predictors for Complications

Parameter	Sig	OR	95% CI	
			Lower	Upper
ASA IV (vs. ASA I)	< 0.001	5.676	3.712	8.680
Anticoagulation (vs. no thromboembolism prophylaxis)	< 0.001	2.433	1.571	3.770
ASA III (vs. ASA I)	< 0.001	2.415	1.874	3.113
Antibiotics as therapy (vs. no antibiotics)	< 0.001	2.096	1.533	2.866
Public insurance (vs. private)	< 0.001	1.781	1.486	2.135
Antibiotic prophylaxis after incision (vs. no antibiotics)	0.046	1.773	1.009	3.115
Thoracic injury (vs. any T code)	0.016	1.687	1.103	2.578
Head injury (vs. any T code)	n.s	1.645	0.989	2.735
Thromboembolism prophylaxis (vs. no thromboembolism prophylaxis)	0.048	1.483	1.003	2.192
ASA V (vs. ASA I)	n.s	1.428	0.161	12.640
Antibiotic prophylaxis before incision (vs. no antibiotics)	0.014	1.417	1.074	1.870
Hip and thigh injury (vs. any T code)	0.002	1.387	1.124	1.710
Sex (male)	< 0.001	1.349	1.164	1.563
Number of operations per case	< 0.001	1.258	1.200	1.318
ASA II (vs. ASA I)	n.s	1.236	0.984	1.553
Intubation yes	n.s	1.154	0.860	1.549
Abdominal, lumbosacral, LWS, pelvis injury (vs. any T code)	n.s	1.089	0.743	1.598
LOS preoperative	< 0.001	1.047	1.028	1.067
Age	< 0.001	1.022	1.018	1.027
ICU duration	< 0.001	1.005	1.003	1.006
Duration surgery	< 0.001	1.002	1.002	1.003
Junior consultant (vs. senior consultant)	n.s	0.972	0.826	1.144
Resident (vs. senior consultant)	n.s	0.849	0.692	1.042
Shoulder and upper arm injury (vs. any T code)	< 0.001	0.559	0.420	0.742
Knee and lower leg injury (vs. any T code)	< 0.001	0.463	0.359	0.595
Elbow and forearm injury (vs. any T code)	< 0.001	0.348	0.254	0.478
Ankle and foot injury (vs. any T code)	< 0.001	0.291	0.152	0.556
Wrist and hand injury (vs. any T code)	< 0.001	0.078	0.019	0.320

*ASA* American Society of Anesthesiologists classification system, *n.s.* not significant, *LOS* Length Of Stay, *ICU* Intensive Care Unit

## Discussion

In the United States, disparities have been reported in treatment and outcome of trauma patients due to unequal insurance status. In Switzerland, basic healthcare is universal, but patients may optionally pay for private insurance. One Swiss study showed less favourable outcomes in publicly insured patients [15].

In this study, we focused on in-hospital mortality and complications. After analysing 30,175 orthopaedic trauma patients, we found publicly insured patients to be younger, more often male, and in better general health according to their ASA physical status. Public insurance was independently associated with complications but not with mortality. Significant predictors for mortality or complications were higher age, male sex, higher (worse) ASA physical status classification, and certain injuries (head, hip, and thigh for mortality; chest, hip, and thigh for complications). We found insurance status to have no effect on length of stay and number of operations per hospitalization.

Our study has several strengths and limitations. One of its strengths is the large sample size with data from multiple institutions. Another strength is the AQC database whose input is voluntary and prospective. This means there are no incentives for data input. The limitations in this study are the following: first, data from the AQC database only account for in-hospital outcomes. Long-term information could not be assessed. Second, our analysis relies on the accurate documentation that physicians entered and therefore lacks validation. Third, the database and ICD-10 classifications give us no information about trauma mechanisms and total injury severity, thereby preventing better risk adjustment analysis. Fourth, the AQC database has missing data in certain areas, especially in procedure codes. For that reason, we could not add this important covariate.

In this study, we found publicly insured patients to be more often male and younger as well as being in better general health (according to ASA physical status categories). These findings are unsurprising and in line with a single-centre study from Switzerland by Jentzsch et al. [15] about the effects of insurance status on trauma patients. They are also congruent with overall fracture age and gender distribution curves published by Court-Brown and Caesar [23].

Patients with private insurance died significantly more often with a rate of 1.9% compared to publicly insured patients with a rate of 1.3%. These findings suggest a relationship between private insurance and mortality, but after adjusting for confounders, neither private nor public insurance could be identified as a significant independent risk factor for mortality. These findings may reasonably be due to the fact that privately insured patients were older (61 vs. 53 years) and in worse general health according to their ASA physical status category. This hypothesis is underscored by several investigations showing that pre-existing health impairments have a significant negative influence on trauma patient outcomes, regardless of injury severity [24, 25]. Additionally, severe injuries involving the head, chest, hip, and thigh were more common in the private cohort of our study.

Literature comparing insured versus uninsured trauma patients has reported that predominantly uninsured patients had higher mortality rates [6–13]. Only one study by Taghavi et al. [26] disagreed and reported that they did not find a correlation between insurance status and mortality. Reasons for higher mortality, such as fewer resources directed to uninsured patients, are critically discussed rather than empirically confirmed [6, 27]. Another possible reason is that insurance status may serve as a surrogate for other factors, such as socioeconomic status (SES). Lower SES has been linked to worse outcomes after non-fatal injury [28], and an association between SES and mortality has been discussed. Yet, a recent study denied a significant association between SES and mortality [29]. These contradictory findings and our results regarding mortality support the thesis that trauma care is often initiated without knowing the patient’s insurance status and therefore leads to comparable treatment and outcomes.

The main predictors for complications were pre-existing medical conditions, need for anticoagulation, distinct injuries (such as chest and head injuries), and public insurance. Patients with public insurance more often had intraoperative and overall complications. The reasons are not yet clear. Longer duration of surgery was a comprehensible independent risk factor for complications. This could be due to the fact that publicly insured patients were more often operated by less experienced surgeons, but after adjusting for confounders, less experienced surgeons were not a predictor for complications. Another possible explanation could be that publicly insured patients were mainly attended by residents in contrast to residents and consultants in the case of privately insured patients. Also, other insurance-associated variables like hospital wealth, qualifications of the attending staff, nursing ratio, and pre-injury nutrition could have an effect on complications that develop throughout the hospital stay. However, this information is not provided in the database and cannot be verified.

Another reason for higher complication rates in publicly insured patients may be the injury severity. In our study, we could not assess for injury severity, so this remains just another potential explanation. Other literature comparing insured versus uninsured patients found injury severity not to differ between the two groups [7, 9, 26]; a study by Salim et al. [10] even indicates that uninsured patients had less severe injuries.

The higher complication rate in publicly insured patients is in contrast to Jentzsch et al. [15] who found no difference in complications between publicly and privately insured Swiss patients. This contrast may be due to their smaller sample size (6000 vs. 30,000) or to a different patient population, as their study included only data from a level-one trauma centre. Another Swiss study on colorectal surgery also found no difference in complications between patients with state versus private healthcare insurance; however, the use of minimally invasive techniques was different in publicly and privately insured patients [30]. We must be aware that this data do not allow to control for a possible effect of over-treatment in patients with private insurance and to reassess the indications – at least the number of operations and length of stay were not significantly different in both insurance groups.

## Conclusion

Differences exist with respect to patient and procedural characteristics in publicly versus privately insured patients. Public insurance is associated with younger age, male sex, and better ASA physical status scores. Insurance status does not seem to be a predictor of fatal outcomes after trauma; however, publicly insured patients are more often subject to complications. These findings need to be interpreted in another country with a mandatory health insurance system.
